# Retiform purpura as the clinical indicator of vascular occlusion after intra-articular knee viscosupplementation

**DOI:** 10.1016/j.jdcr.2026.05.004

**Published:** 2026-05-12

**Authors:** Thomas Brenden Whiting, Grishma Patel, Mark Lewis, Nisha Patel, Jacqueline Hwang, Jean Margaret Moresi, Dakarai Dunbar, Aisha Mumtaz

**Affiliations:** Department of Dermatology, University of Maryland School of Medicine, Baltimore, Maryland

**Keywords:** durolane, hyaluronic acid injection, intra-articular hyaluronic acid, livedo reticularis, orthopedic vascular occlusion, osteoarthritis viscosupplementation, retiform purpura, vascular occlusion

## Introduction

Hyaluronic acid (HA) injection is generally well tolerated in cosmetic dermatology and in musculoskeletal medicine for intra-articular viscosupplementation.^1^^,^^2^ Durolane is a non-animal, biofermented form of HA stabilized via cross-linking.^1^

HA-related Vascular Occlusion (VO) in cosmetic settings occurs in 0.05% to 0.1% of cases.^3^ Reports following intra-articular HA injection are exceedingly rare and its incidence remains unknown.^3^

VO clinically presents as immediate painless blanching followed by livedo reticularis. Later, retiform purpura with progression to pustules, slough, or necrotic eschar may develop.^4^^,^^5^ In cases of HA-induced VO, histologic identification of the HA filler can be made using alcian blue stain at pH 2.5 to highlight acidic mucopolysaccharide deposits from punch biopsy specimens.^6^ These deposits appear as amorphous basophilic material on H&E and are PAS negative.^6^

We report a case of VO secondary to intra-articular HA injection. This case highlights a dermatologic diagnosis of a rare orthopedic complication—illustrating the need for multidisciplinary collaboration, early recognition, and development of treatment protocols to guide intervention.

## Case presentation

A 49-year-old male presented to dermatology 13 days following intra-articular HA injection. Empirical diagnosis of retiform purpura with early necrosis and pustule formation prompted high clinical suspicion for VO. Resolution at 14 weeks followed a course of initial livedo reticularis evolving into retiform purpura with necrotic slough, pustule formation, ulceration and, ultimately, post-inflammatory hyperpigmentation with dermal fibrosis.

**Fig 1:**
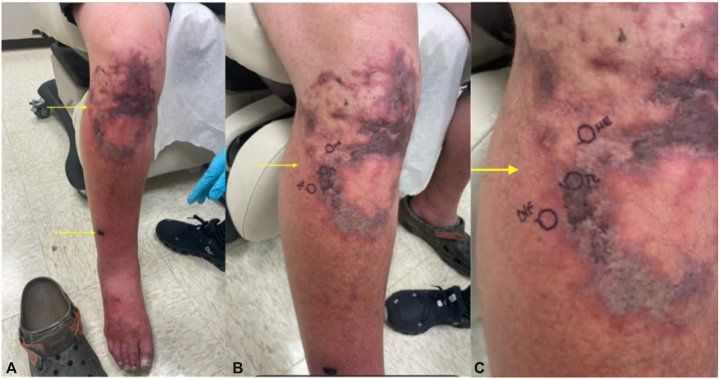
**A,** 13 days post-injection. Right lower extremity with retiform purpura and early necrosis to right knee and necrotic, eroded site of patient-reported pustule to right lateral lower leg. **B,** Biopsy sites labeled “H&E,” “TC,” and “DIF.” **C,** Close-up of biopsy sites labeled “H&E,” “TC,” and “DIF.”

The patient reported the appearance of a purplish, net-like discoloration over the anterior knee immediately following injection and, within days—edema, erythema, violaceous darkening, and hyperalgesia. At day 13, dermatologic evaluation identified fixed, branching, violaceous patches with central darkening and skin breakdown as well as an eroded, fluid-filled vesicle that had reportedly released clear fluid (Fig 1).

Diagnostic workup and punch biopsies ruled out differential diagnoses of vasculitis or of depositional, infectious, thrombotic, and embolic pathology, leading to strong clinical suspicion of VO.^7^ The following blood studies were within normal limits: complete blood count (CBC), complete metabolic panel (CMP), D-dimer, C-reactive protein (CRP), antinuclear antibody (ANA), rheumatoid factor (RF), complement levels (C3), and immunoglobulin G (IgG). Blood cultures were unremarkable. Duplex ultrasonography ruled out deep vein thrombosis. Computed tomography (CT) of the lower extremity demonstrated non-specific subcutaneous edema. CT angiogram showed small anastomoses suggestive of neovascularization, supporting clinical suspicion of VO.

**Fig 2:**
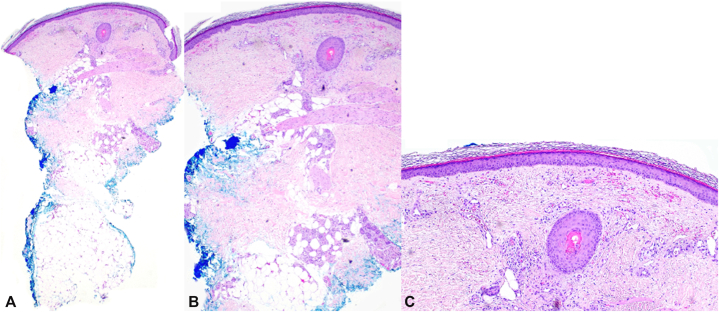
Dermatopathology diagnosis with comments: “Mild upper dermal proliferation of small vessels and extravasated erythrocytes. Initial and multiple levels sections are examined. A sparse inflammatory infiltrate comprised of mononuclear cells is present. There is no evidence of fibrin thrombi or vasculitis, nor is evidence of calciphylaxis seen. No fungal hyphae are identified on PAS-F stain. (Appropriate controls were reviewed.) Clinicopathologic correlation is recommended.” **A,** 2× view of hematoxylin-eosin (H&E) staining from punch biopsy of right knee. **B,** 4× view of hematoxylin-eosin (H&E) staining from punch biopsy of right knee. **C,** 10× view of hematoxylin-eosin (H&E) staining from punch biopsy of right knee.

Three punch biopsies were obtained for H&E staining, tissue culture, and direct immunofluorescence (DIF) (Fig 1). Histopathological studies showed no fibrin, thrombi, vasculitis, or calciphylaxis. Periodic acid-Schiff for fungus (PAS-F) staining, tissue culture, and DIF were negative. Dermatopathology report was negative for both vasculopathy and diagnostic HA features of acidic mucopolysaccharide deposits on hematoxylin-eosin (H&E) (Fig 2). Alcian blue staining at pH 2.5 was thus not pursued.

**Fig 3:**
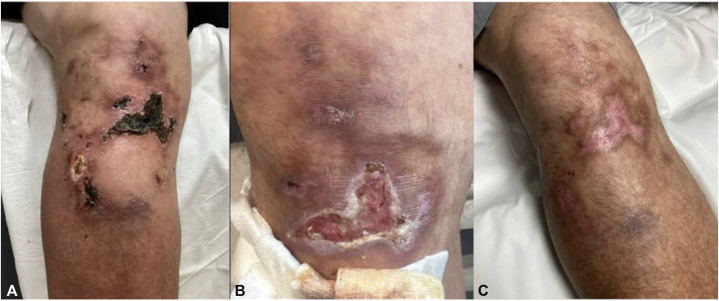
**A,** 46 days after injection showing necrotic slough and early ulceration with surrounding hyperpigmentation to right knee. **B,** 75 days after injection showing ulceration and surrounding hyperpigmentation to right knee. **C,** 105 days post-injection showing residual hyperpigmentation and scar formation to right knee.

After extensive exclusion of differential diagnoses and multidisciplinary evaluation by infectious disease, vascular surgery, emergency medicine and orthopedics, the case was clinically confirmed as VO. A multidisciplinary treatment approach was initiated during this acute complication. Multiple antibiotic courses of amoxicillin-clavulanate, doxycycline, linezolid, as well as non-steroidal anti-inflammatory drugs (NSAIDs), antihistamines, and a steroid taper failed to significantly improve symptoms. Although patient compliance was limited, topical nitroglycerin paste massage and compression stockings were advised. Conservative management, including Xeroform, MediHoney, Hydrofera Blue and gabapentin for pain, preceded discharge by wound care without contracture or other extenuating complication at week 14 (Fig 3).

No invasive intervention was pursued given delayed recognition by primary orthopedic team, stable disease course, and cross-specialty uncertainty regarding this complication. Over months, the purpura and necrosis resolved into hyperpigmentation and fibrosis (Fig 3).

## Discussion

Similar mechanisms may underlie complications following cosmetic use of HA although few reports describe VO following intra-articular HA. The clinical evolution, together with extrapolated treatment principles from cosmetic VO, suggests that an initially reversible perfusion disturbance progressed to irreversible ischemia and tissue injury.

The presence of painless, non-blanching skin and livedo reticularis immediately post-injection represents a critical diagnostic and narrow therapeutic intervention window in HA-related vascular compromise.^4^^,^^5^ Future protocols may include monitoring of skin changes in orthopedic HA injections for timely administration of hyaluronidase, vasodilators, anticoagulants, or other interventions like hyperbaric oxygen.^6^

Although direct evidence in joint or periarticular settings is lacking, these interventions collectively target the same pathophysiologic cascade—mechanical obstruction, microvascular thrombosis, and progressive tissue hypoxia—and may plausibly mitigate downstream necrosis if instituted promptly. A similar case that exhibited progressive sequelae following intra-articular injection, with histologic confirmation, described the first reported use of intradermal hyaluronidase without associated adverse effects. Although treatment appeared to halt further progression of symptoms, it was administered 22 days after the initial injection, and the overall time to resolution was comparable to cases without intervention.^6^

No standardized protocol for intra-articular HA-related VO exists. While it is reassuring that reported cases generally resolve with conservative management across months, there is still risk of contracture or other sequelae. Thus, early identification and treatment, potentially mirroring cosmetic vascular occlusion monitoring and interventions, may diminish severity of complications and shorten recovery timeframes. Multidisciplinary collaboration and broader awareness are essential to develop workup and intervention protocols to support early recognition and management of complications. Furthermore, this case illustrates that absence of histologic confirmation should not delay intervention when clinical suspicion is high.

### Declaration of generative AI and AI-assisted technologies in the writing process

During the preparation of this work the author(s) used ChatGPT to edit the manuscript for grammar, structure, and clarity. After using this tool/service, the author(s) reviewed and edited the content as needed and takes full responsibility for the content of the published article.

## Conflicts of interest

None disclosed.
